# Supplementary material for: “Hurts less, lasts longer”; a qualitative study on experiences of young people receiving high-dose subcutaneous injections of benzathine penicillin G to prevent rheumatic heart disease in New Zealand
Source: PLoS One. 2024 May 14;19(5):e0302493. doi: 10.1371/journal.pone.0302493 (PMC11093343; doi:10.1371/journal.pone.0302493)
Supplement: Attachment — Submitted filename: SCIP II Review.pdf [file pone.0302493.s002.pdf]

“Hurts less, lasts longer”: experiences of young people receiving high-dose subcutaneous infusions of benzathine penicillin G to prevent rheumatic heart disease.

Overall Summation:

This study describes a welcome and important innovation considering the length of time that the only option for secondary prophylaxis has been via deep intramuscular injection, with its associated problem of pain. The paper reports on the qualitative component of a Phase-II medication trial. It explores experiences of the new medication formulation among the recruited participants.

The importance and innovative nature of this study is currently let down by its non-alignment with standards in the reporting of qualitative research, such as SRQR. A review to align the paper with guidelines will greatly improve it and ensure that readers perceive that the study is rigorous, believable, and generalisable.

The study was conducted with participants who are Indigenous and, in this case, are impacted by a condition known to arise in settings of socioeconomic disadvantage, power and opportunity within a nation boasting a high standard of living, Aotearoa. The need to use Indigenous methodologies in conducting studies in such settings is a given. I am sure that the study was conducted ethically in this regard, but the current writing does not demonstrate this well enough to the reader. The researchers have utilised Kaupapa principles. This has been given a “passing nod” that is not convincing and is one place where the paper should be strengthened. As recommended in SRQR guidelines, the Indigenous status of researchers, including those who conducted interviews and observations needs to be made clear.

Pain and frequency of the usual SP regimen are presented as the only limiting factors impacting adherence to the injection regimen. As I am sure the authors are aware, these are just some of the limiting factors. See synopsis for a more accurate description to do with adherence.

One of the strongest outcomes of this trial is the potential to provide another SP option for patients to choose from. Query as to what happened at the end of the trial to the 19 participants who strongly preferred SCIP needs to be included.

Title:

The title should clearly show the genre of the research and the study site.

Suggestions:

“Hurts less, lasts longer”: a qualitative study on experiences of young people receiving high-dose subcutaneous infusions of benzathine penicillin G to prevent rheumatic heart disease in Aotearoa (or New Zealand).

Or

A qualitative study on experiences of young people receiving high-dose subcutaneous infusions of benzathine penicillin G in a Phase-II trial to prevent rheumatic heart disease in Aotearoa (or New Zealand): "hurts less, lasts longer".

Abstract:

Line 7 Methodology:	Need to include "methodology". Currently just states methods (interviews and observations- and the trial process).<br>Suggest adding at the start of this paragraph:<br>"This study used Kaupapa principles to explore Māori and Pacific young peoples' experiences."
Line 17	Remove the emotive word "overwhelmingly" to fit with academic writing style.
Line 19	Suggest replacing "treatments" with injections.<br>No matter which method patients choose, it is still an injection.

Synopsis:

In general,	A solely biomedical description of ARF and RHD in a qualitative paper. This likely reflects the fields of expertise of most of the authors. Suggestions below
Line 25	Suggest inserting the following text at the end of the sentence after the word infection:<br>"…predominantly among people living in socioeconomic disadvantage."
Line 29	Needs a more accurate description to do with adherence problems. Change sentence to:<br>"The effectiveness of this approach is limited by complex issues including the young age of patients and health system frailties *(as in your ref 8).* However, pain, fear, and the frequency of injection are recognised as common barriers to the injection adherence."
Line 34	Replace "overwhelmingly" with strongly
Line 35-36	Put the number 70 first to match with the preceding text

Introduction:

Line 48	"…for Indigenous Māori".<br>Debates exist around use of this term in Aotearoa. Could distract some readers. Note that in ref 4, some of the same authors as in this paper use the phrase "people of Māori and Pacific Islander ethnicity".
Line 58	Typo- recommend should be recommended
Line 62	Change beginning of sentence to:<br>"Pain and fear associated with IM……."

Line 67	New formulations are urgent but adherence to the regimen is more complex than just injection pain and there is an equally urgent need, for instance, to improve housing in NZ for Māori and Pacific people to prevent ARF.<br>Suggestions for new sentence:<br>However, to improve adherence and prevent disease progression, new formulations of long-acting penicillins are warranted.<br>or<br>Improving the formulation of long-acting penicillin has potential to improve adherence and prevent disease progression.
Line 73 and 74	For later ref in last paragraph of the Discussion, need to mention the global experts' conclusions about cold chain and cost mentioned here.
Line 79	End of sentence change to "no significant adverse effects"
Line 89 and 90	Note: These lines show the additional measures that should be available to address pain and fear of injection episodes for young people thus providing evidence that the authors do know that the injection experiences are more complex than pain alone.
Line 97	Suggest change to:<br>This paper reports on the qualitative component of the SCIP-II trial aiming to explore participants' experiences of SCIP compared with the usual intramuscular regimen.

Methods

Further information that aligns with the Kaupapa principles is needed in describing recruitment of participants.

Line 93	Suggested rewrite:<br>"In brief, participants with ARF, were identified through a regional register of secondary prophylaxis. X were invited to be part of the study by attending an outpatient clinic (designated for the study?) in Wellington, NZ."<br>A brief statement of how the participants were invited is needed.<br>Were they contacted by phone, email or in person? And by whom? How were explanations of the study provided? This info is needed to demonstrate a Kaupapa approach of respecting participants' equal power to choose to take part in the study or not.<br>Finally,<br>"Twenty participants agreed to participate in the study which ran from 21st November 2022 until 20th March 2023. Participants were followed for 70 days after their initial clinic visit where they received SCIP."
Table line 106	For clarity, change "Number of vials" to "Number of prefilled syringes"
Line 110	Change data collection here to a new section heading, "Methodology".<br>This new Methodology section would assist flow if it were placed before the Methods section because it provides rationale for why certain methods were undertaken.

	Stating the methodology strengthens qualitative papers and is one indicator of quality of a study. Not being clear about methodology is a common trap in qualitative components of drug trials.<br>Suggested text below

Methodology

Line 111	Suggested example to replace existing text on Kaupapa principles in research. Current explanations of Kaupapa in the paper are vague and unconvincing for the reader:<br>"This qualitative study used Kaupapa Māori research principles as a framework. These principles prioritise Māori perspectives and wellbeing by attending to unequal power relations and rejecting deficit explanations."<br>This is where you need to state that the study involved Māori or Pacific researchers, nurses of assistants.<br>How were participants given feedback on results? Did they know upfront that it was a trial and that SCIP would not be available after the study ended? How did you mitigate a sense of being experimented on?
Line 116	This subheading of "data collection" describes methods. Suggest moving it to Methods section, below the table 1. Commence with Participant observations….<br>Note, interviews and observations are methods, not methodology.<br>Text to do with Kaupapa now under Methodology.
Line 120	All interviews occurred face to face by whom? Initials of researcher suffice.<br>I note in the ethics section that consent forms were available in Te Reo Māori and Samoan. This implies that language was an issue?? What languages were the interviews conducted in? Were interpreters needed or used?
Line 122	Observational data were collected by the study nurse or researcher. Insert their initials. Hopefully we know that they are Māori or Pacifica or not by this point in the paper to reflect Kaupapa principles and transparency.
Line 127	Ambiguous and needs more detail about:<br>'a community nurse' what is her role and involvement with SP or other?<br>'two researchers': which ones? Were they clinicians, qualitative researchers, authors on this paper? Convince the readers that these interviews were relevant and added value.
Line 129	Remove 'quantitative', the pain was measured quantitatively.<br>Quantitative is implied in the scoring system used.<br>Qualitative studies can also include quantitative methods.
Line 132	Insert "a reduction of" 1.3 and 1.8 respectively….

Data analysis

Line 137	Change to: Interviews and field observations were transcribed verbatim for analysis.

Line 138	Transcripts were read repeatedly by [insert initials] and remove "one investigator"
Line 139	Thematic saturation is a given in qual research. This sentence and the following sentence could be removed. Better to state that there was early congruence in themes arising from transcripts.
Line 142	Here we finally hear that the study nurse, a community nurse, and two researchers were present during infusions. This info needs to come in line 127. This sentence not needed here as it is previously explained that they were interviewees.
Line 144 and 145	This is an important sentence that needs further information to verify the Kaupapa framework. The codes and themes were presented to Māori and Pacific researchers (the first time we hear such researchers were involved). We need to hear their responses. Did they adjust any codes, make suggestions, provide insight into cultural aspects? Additionally, was any feedback given to participants? Did this lead to any changes in themes?

Ethical approval

Line 153	Were the consent forms also available in English?

Results

Lines 159 and 160	Age range states 6-32 but table has youngest as 7 years.<br>Suggest remove "Indigenous"

Thematic analysis

Line 165	Suggest: "Overall, the thematic analysis………<br>Some initial anxiety from participants to with… what? Fear of unknown? Dealing with unfamiliar practitioners?
Line 171	Change to "Anxiety prior to SCIP" or "Anxiety in anticipation of SCIP"
Line 172	How was anxiety evident? "Anxiety was either stated or observed in many participants."
Line 189	…short, sharp or caused a stinging sensation that quickly subsided.
Line 196	…participants described having no pain or minimal pain during……
Line 212	Tautology: don't need small with minority
Line 216	"Two participants experienced some distress and discomfort, with corresponding pain scores of seven." Without a prior understanding of ……
Line 223	Move this sentence to Discussion.
Line 231	"…had resumed usual activities."
Line 255	Change heading to: Strong preference to remain on three-monthly SCIP rather than BPG
Line 257<br>Line 258	No need for brackets<br>Suggest change to:

	"The study nurse observed participants' sense of relief at the prospect of being able to change from IM BPG to SCIP".<br>This is why readers need to know if it was explained to participants at the start of the study if ongoing SCIP would be an option at the end of the study. It was predictable that if a strong preference emerged for SCIP that this question about continuing SCIP would arise among participants.
Line 266	"…who tolerated SCIP well

Discussion

Line 273	Replace overwhelmingly with strong
Line 276	Suggest change to:<br>"The seven-year-old participant who chose a known provider over SCIP highlights the importance of provider relationships and child-focussed care for children undergoing repeated painful procedures such as SP" (ref new Australian RHD guidelines).
Line 279	…due to 'reported' lower pain levels…<br>Pain is still a subjective experience
Line 282	Replace reinforce with support
Line 286	Replace last sentence:<br>This meant that participants had less interruption to their usual work or school schedules to obtain injections and increased their freedom to travel.
Line 289	Suggest change to:<br>"Administering SCIP through a series of slow pushes enabled more control in the rate of delivery in comparison to the ……"
Line 301	Change to…we did not ask about "participants usual or general pain tolerance prior to the study".
Line 302	Remove the words "and contrast"
Line 304 to 309	These sentences are a red flag to qualitative researchers.<br>As it currently reads, there are negative assumptions that I doubt the authors intend about some participants and which defy Kaupapa principles.<br>Please review these sentences with Māori researchers for insight and clarity.<br>For instance, were the difficulties due to language or cultural barriers or unknown interviewer?
Line 313	Suggest addition:<br>"…. participants were able to discuss life issues as well as challenges with regular IM BPG indicating their preference for holistic approaches in healthcare delivery"
Line 314 to 316	I think you mean that use of the Kaupapa approach as an Indigenous methodology helped participants to feel empowered and treated as partners rather than subjects of research.<br>This sentence needs changing.
Line 318 to 324	This study reports a great breakthrough in SP. However, we need to be mindful that the current formulation of SCIP is only possible where there is a cold chain and where it can be afforded. This indicates that more work needs to be done for those sites that don't have these advantages. Suggest a reword:

	"SCIP utilising prefilled syringes is preferred over IM BPG as a tolerable and acceptable route of administration for children and young adults with ARF in Wellington, NZ. Extending this study to support widespread choice of SCIP throughout NZ for patients requiring secondary prophylaxis over a prolonged period will enable better assessment of key measures over time. We note that SCIP does not yet align with all recommendations of global experts in that it should be cold chain independent and of comparable cost for greatest global benefit. It would be useful to undertake an economic evaluation of SCIP in comparison to IM BPG. Further work on cold chain independence is warranted. Current evidence suggests that SCIP will likely become the standard of care for most patients needing long-term prophylaxis. SCIP has the potential to improve patient experiences which may assist prevention of disease progression and death, transforming prophylaxis of ARF/RHD both in NZ and globally.
